# Transversal Displacement Detection of an Arched Bridge with a Multimonostatic Multiple-Input Multiple-Output Radar

**DOI:** 10.3390/s24061839

**Published:** 2024-03-13

**Authors:** Lorenzo Pagnini, Lapo Miccinesi, Alessandra Beni, Massimiliano Pieraccini

**Affiliations:** Department of Information Engineering, University of Florence, 50139 Firenze, Italy; lorenzo.pagnini@unifi.it (L.P.); alessandra.beni@unifi.it (A.B.); massimiliano.pieraccini@unifi.it (M.P.)

**Keywords:** bridge monitoring, displacement vector, interferometry, MIMO, multimonostatic radar, vibration measurement

## Abstract

Interferometric radars are widely used for monitoring civil structures. Bridges are critical structures that need to be constantly monitored for the safety of the users. In this work, a frequency-modulated continuous wave (FMCW) multiple-input multiple-output (MIMO) radar was used for monitoring an arched bridge in Catanzaro, Italy. Two measurements were carried out; a first standard measurement was made in a monostatic configuration, while a subsequent measurement was carried out in a multimonostatic configuration in order to retrieve the components of the deck displacement. A method that is able to predict the measurement uncertainty as a function of the multimonostatic geometry is provided, thereby aiming to facilitate the operators in the choice of the proper experimental setup. The multimonostatic measurement revealed a displacement along the horizontal direction that was four times higher than the one along the vertical direction, while the values reported in the literature correspond to a ratio of at most around 0.2. This is the first time that such a large ratio detected by radar has been reported; at any rate, it is compatible with the arched structure of this specific bridge. This case study highlights the importance of techniques that are able to retrieve at least two components of the displacement.

## 1. Introduction

Structural monitoring plays a key role in deterioration detection and damage prevention. In fact, relevant information retrieved from monitoring activities can be used to optimize maintenance interventions in a cost-effective way. In the last twenty years, monitoring techniques have been deeply investigated. Different sensors are still being studied for this purpose depending on the structure under test. Bridges are civil structures whose monitoring is extremely important in terms of safety; great effort has been made in this context, as witnessed by the literature [1,2,3,4]. Several parameters need to be detected for bridge health, including strain, acceleration, displacement, temperature, and inclination [5]. For what concerns the displacement, contact sensors such as linear voltage differential transformers [6], ultrasonic sensors [7,8], ultrawideband (UWB) sensors [9], and accelerometers [10,11,12] provide high accuracy and are light and easy to maintain. However, their main limitation comes out when areas with difficult access have to be monitored. Noncontact sensors such as lasers [13,14,15,16] and radars [17,18,19,20] make it possible to overcome this limitation. In the case of high-bridge monitoring, the limited operating range of the lasers leads the radars to be the best candidate.

In the last years, radars have been of great interest in this context. Frequency-modulated continuous wave (FMCW) radars detect the target by performing a comparison between the transmitted signal and the received signal by a mixer [21]. For retreiving the displacement, the phase information of the signal related to the target is employed; this technique is known as interferometry. Typically, the radar is installed under the deck of the bridge, and the displacement is assumed along the vertical direction. However, Dei et al. [22] experimentally showed how the detection of a single component can be misleading. To solve this problem, some solutions were proposed in the last years.

In 2015, Li et al. [23] analyzed theoretically the 3D displacement; the purpose of their work was to investigate the simultaneous measurement of multiple targets. In 2018, Monti-Guarnieri et al. [24] simultaneously operated two radars to detect the displacement of an industrial pipeline, and great effort was made for the clutter removal. Deng et al. [25] deployed three ground-based radars to measure the 3D deformation of a movable corner reflector. The monitoring was carried out statically, and a method for the evaluation of the uncertainty of the displacement direction was proposed. In [26], two radars were employed in bridge monitoring. In order to highlight the importance of using more than one sensor, an analytical formula concerning the error in the vertical component in the case of a single radar is provided. In [27], the problem of removing external disturbances from the measurements of three interferometric radars was addressed. Moreover, a qualitative discussion of the measurement uncertainty depending on the geometry was carried out. In [28], the uncertainty related to the clutter and the geometry in the case of using two radars was deeply investigated. However, the main limitation of all the above works relies on the fact that a synchronization a posteriori was needed, which inevitably introduces uncertainty.

Great attention has been devoted to multiple-input multiple-output (MIMO) radars in recent years, such as in satellite applications [29], ground-based radar [30,31,32] and also in the biomedical context [33]. In contrast to these works where the MIMO feature was exploited for a better angular resolution, in 2021, Miccinesi et al. employed it to achieve two independent radars [34,35]; the detection of two displacement components of a bridge was performed, thus overcoming the synchronization issues. The multimonostatic technique implemented in these works used a radar and a transponder to monitor the infrastructures from two different locations whose distance is called the baseline. The transponder can be connected to the radar through radio frequency (RF) cables or with a radio link; the latter option makes it possible to overcome the physical limits related to the length of the RF cables. In [34], the radar and the transponder were placed at a different quote, and the link was cable-based. In [35], the radar and the transponder were approximately at the same quote, and the link was radio-based. This link made it possible to increase the baseline by up to ≈100 m with respect to the cable-based one but at the cost of a considerable effort in terms of the complexity and installation time.

In the present work, a first measurement was carried out in the standard monostatic configuration. Subsequently, a second measurement was carried out in the multimonostatic configuration. In the latter configuration, the radar and the transponder were placed at the same quote and the link was cable-based, thus making the measurement setup straightforward. The main purpose of the work was to detect the vertical and horizontal components of arched bridge displacements.

**Figure 1:**
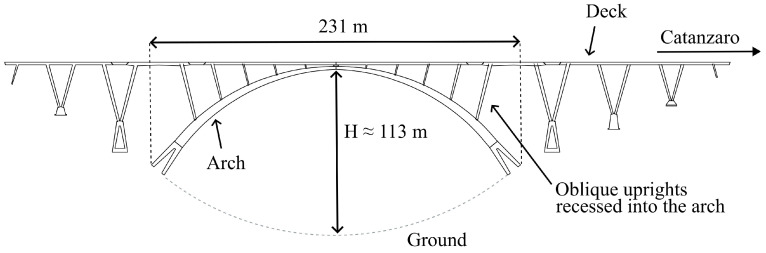
Draft of Bisantis bridge in Catanzaro, Italy.

The bridge’s name is Bisantis; it is located in Catanzaro, Italy, and it was designed by the best-known Italian civil engineer Riccardo Morandi in the early sixties. Over the same years, the engineer also designed the Polcevera bridge, which fell in 2018. After its tragic collapse, synthetic aperture radar (SAR) data collected from satellites up to 2018 were used to detect possible precursors [36,37,38]. Similar SAR techniques were applied to the Bisantis bridge, thereby leading to the detection of the vibration amplitude pattern of the deck [39]. The Bisantis bridge is an arched bridge made by reinforced concrete: the horizontal deck is connected to the arch by some oblique uprights. The first design of the bridge included two central support pillars; however, this was later avoided because the foundation soil at the bottom of the valley was alluvial, while that of the walls was solid rock. A draft of the bridge is shown in Figure 1. The bridge is about 113m high with a maximum span of 231m. The width is 12.5m, and the length is 468.25m. Due to the arch, this bridge is extremely rigid in the vertical direction, while it should have a large displacement component along the transversal direction on the horizontal plane due to the strong wind blowing in the gully during the measurement sessions.

## 2. Materials and Methods

### 2.1. Data Processing Methods

As previously mentioned, the interferometric radar uses phase information to retrieve the displacement of the target in its field of view. The acquired echo is a complex number $E_{n,m}$, where $n\in [1,2,\ldots ,N_{sample}]$ is the fast time index, and $m\in [1,2,\ldots ,N_{sweep}]$ is the slow time index; $N_{sample}$ is the number of samples per sweep, while $N_{sweep}$ is the total sweep per measurement. The fast Fourier transform (FFT) along the fast time of $E_{n,m}$ is calculated to obtain the information about the distance (R) of the target:

(1) $$ff_{m}\left(R\right)=FFT_{n}\left(E_{n,m}\right),$$

where the amplitude of $ff_{m}\left(R\right)$ corresponds to the point spread function (PSF) of each single target in the field of view.

The phase of $ff_{m}\left(R\right)$ can be used to evaluate the displacement of the target by using the following relationship:

(2) $$\Delta R=\frac{\lambda }{4\pi }\Delta \phi_{m,m+1},$$

where ΔR is the displacement of the target, λ is the wavelength, and $\Delta \phi_{m,m+1}$ is the difference of the phase between two measurements.

The cumulative displacement data can be employed in the joint time frequency analysis (JTFA) [40] for retrieving the natural frequencies of the bridge.

### 2.2. Geometry Description

**Figure 2:**
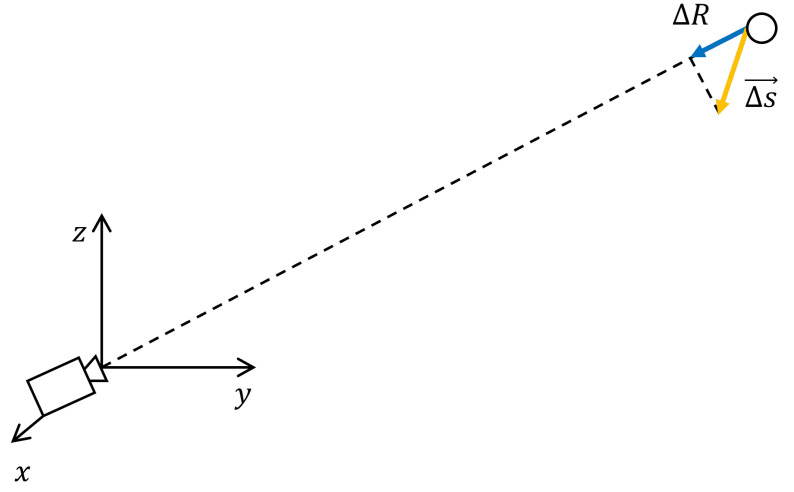
Geometry of a monostatic interferometric measurement.

In the case of the monostatic measurement, the radar was placed under the target as depicted in Figure 2. The displacement ΔR is the projection of the displacement $\Delta \vec{S}$ along the direction of the radar target.

**Figure 3:**
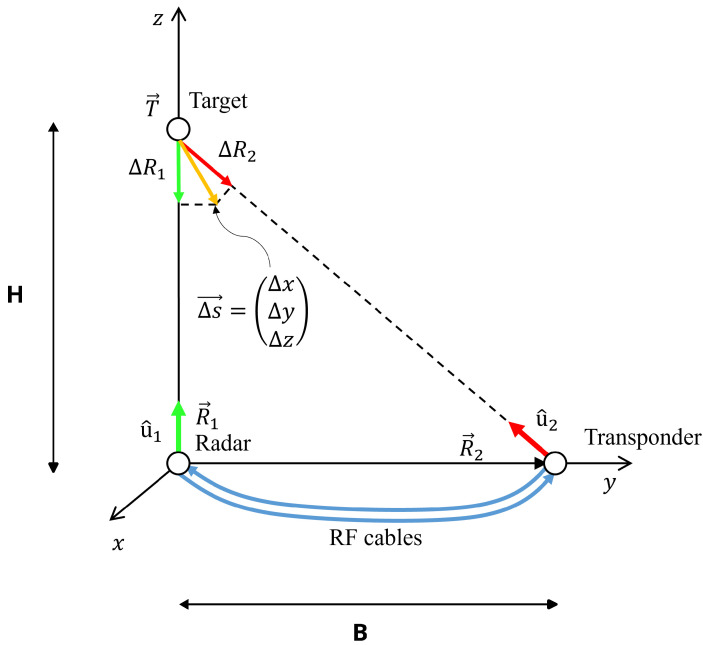
Geometry of a multimonostatic interferometric measurement.

In the case of the multimonostatic measurement, the radar and the transponder were placed under the target as depicted in Figure 3. The displacement components along the vertical and horizontal directions can be calculated as explained below. The unit vector radar target ${\hat{u}}_{k}$ is defined as follows:

(3) $${\hat{u}}_{k}=\frac{\vec{T}-{\vec{R}}_{k}}{|\vec{T}-{\vec{R}}_{k}|},\;k=1,2$$

where $\vec{T}$ is the position of the target, and ${\vec{R}}_{1}$ and ${\vec{R}}_{2}$ are the positions of the radar and the transponder, respectively.

The relation between the components $\Delta R_{1,2}$ measured by the two devices and the Cartesian axis components Δy, Δz is

(4) $$\left(\begin{array}{c} \Delta R_{1}\\ \Delta R_{2} \end{array}\right)=\left(\begin{array}{cc} {\hat{u}}_{1}\cdot {\hat{e}}_{y} & {\hat{u}}_{1}\cdot {\hat{e}}_{z}\\ {\hat{u}}_{2}\cdot {\hat{e}}_{y} & {\hat{u}}_{2}\cdot {\hat{e}}_{z} \end{array}\right)\left(\begin{array}{c} \Delta y\\ \Delta z \end{array}\right)=M\cdot \left(\begin{array}{c} \Delta y\\ \Delta z \end{array}\right),$$

where ${\hat{u}}_{k}$ is given by Equation (3), and ${\hat{e}}_{y}$, ${\hat{e}}_{z}$ are the unit vectors of the coordinate axes. It is important to note that the signal of the two receiving antennas must be acquired at the same time.

By expanding the elements of the matrix *M* and rearranging them as functions of the bridge height *H* and the baseline *B*, one can obtain the following:

(5) $$M=\left(\begin{array}{cc} 0 & 1\\ \frac{B}{\sqrt{B^{2}+H^{2}}} & \frac{H}{\sqrt{B^{2}+H^{2}}} \end{array}\right).$$

Therefore, the two components can be retrieved by inverting the matrix *M*, thus resulting in the following:

(6) $$\left\{\begin{array}{c} \Delta y=-\frac{H}{B}\Delta R_{1}+\frac{\sqrt{B^{2}+H^{2}}}{B}\Delta R_{2}\\ \Delta z=\Delta R_{1} \end{array}\right.$$

### 2.3. Uncertainty Evaluation

**Figure 4:**
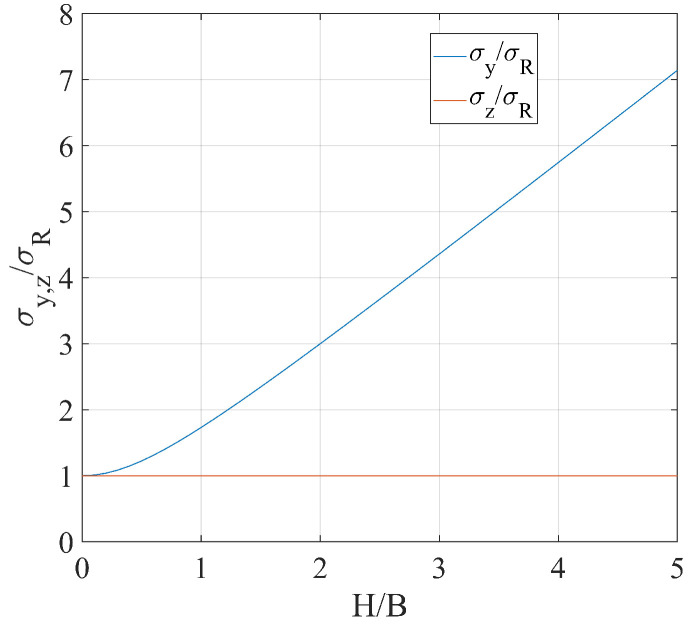
Uncertainty $\sigma_{y}$ normalized with respect to $\sigma_{R}$ as a function of the ratio H/B.

The error propagation formula was applied to Equation (6), thus obtaining the following:

(7) $$\left\{\begin{array}{c} \sigma_{y}=\sqrt{{\left(\frac{H}{B}\sigma_{R_{1}}\right)}^{2}+\left[{\left(\frac{H}{B}\right)}^{2}+1\right]\cdot \sigma_{R_{2}}^{2}}\\ \sigma_{z}=\sigma_{R_{1}} \end{array}\right.$$

where $\sigma_{y}$ and $\sigma_{z}$ are the uncertainties along y and z, respectively, and $\sigma_{R_{1,2}}$ are the uncertainties affecting $\Delta R_{1,2}$. From Equation (7), it is evident that $\sigma_{y}$ depends on the ratio between H and B. The uncertainty was normalized with respect to $\sigma_{R}=\sigma_{R_{1}}=\sigma_{R_{2}}$, and the resulting ratio was plotted as a function of H/B, as shown in Figure 4. For completness, $\sigma_{z}$ was considered as well. For the geometry adopted in this work, $\sigma_{y}>\sigma_{z}$ for each value of H/B. It is worth noting that, in general, the condition H/B=0 could correspond to two different situations, namely H=0,B≠0 and H≠0,B→∞; in this work, the latter situation was considered.

Once the components have been retrieved, it is possible to define the angle of the displacement with respect to the horizontal axis as follows:

(8) $$\alpha =atan\left(\frac{\Delta z}{\Delta y}\right),$$

where the following equation provides a relation between the uncertainties on $\sigma_{R_{1}}$ and $\sigma_{R_{2}}$ and the one affecting α:

(9) $$\sigma_{\alpha }=\frac{1}{\Delta R_{1}^{2}+{\left(-\frac{H}{B}\Delta R_{1}+\frac{\sqrt{B^{2}+H^{2}}}{B}\Delta R_{2}\right)}^{2}}\left[\left|\frac{\left(\sqrt{B^{2}+H^{2}}\right)}{B}\Delta R_{2}\right|\sigma_{R_{1}}+\left|\frac{\left(\sqrt{B^{2}+H^{2}}\right)}{B}\Delta R_{1}\right|\sigma_{R_{2}}\right]$$

where $\sigma_{\alpha }$ is the uncertainty in the evaluation of the displacement direction.

Equation (9) deserves a further investigation as explained below. Given the presence of the terms $\Delta R_{1}$ and $\Delta R_{2}$ on the second member, one can deduce that the uncertainty $\sigma_{\alpha }$ should depend on both α and the displacement module Δs. The uncertainty $\sigma_{\alpha }$ was normalized with respect to $\sigma_{R}=\sigma_{R_{1}}=\sigma_{R_{2}}$, and the resulting ratio was computed as a function of α, with Δs as parameter. The value H/B was set to 5, thus corresponding to the specific geometry of this work.

**Figure 5:**
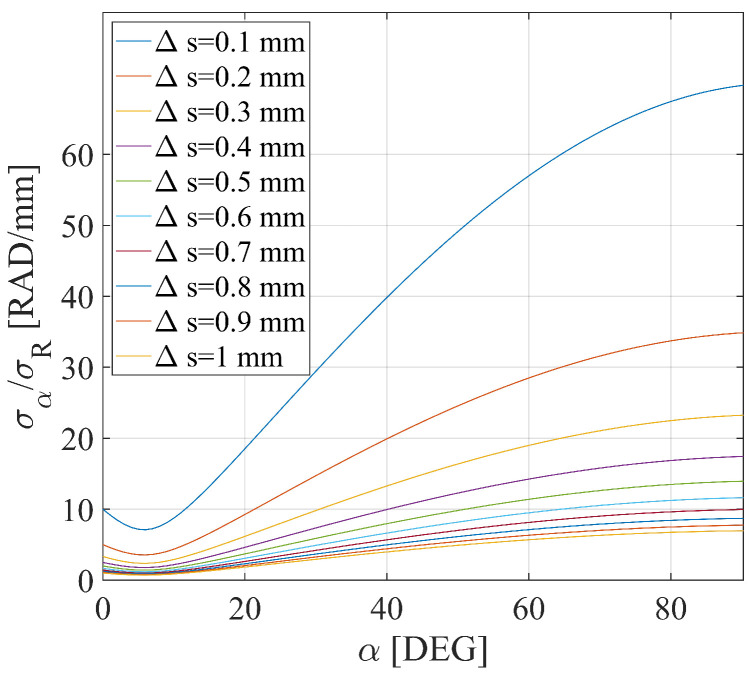
Uncertainty $\sigma_{\alpha }$ normalized with respect to $\sigma_{R}$ as a function of α for different values of Δs; H/B≈5.

**Figure 6:**
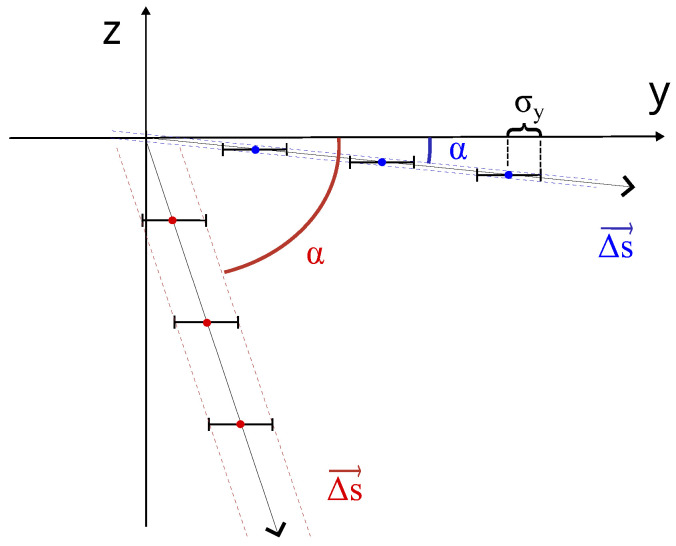
Example of displacements $\vec{\Delta s}$ visualized as scatter plots in the y−z plane. The uncertainty along the horizontal direction, $\sigma_{y}$, leads the vertical displacements (red) to be more dispersed with respect to the horizontal ones (blue). For simplicity, $\sigma_{z}=0$.

The results are reported in Figure 5. Concerning the dependence on α, when the oscillation occurs vertically (α=90°), the uncertainty of the angle is at its maximum, and it tends to decrease as the oscillation becomes horizontal up to the minimum for α≈8°. Similarly, the larger the displacement, the smaller the uncertainty of the angle α. As a first impression, these facts could seem a bit surprising; however, the explanation relies on the fact that for this specific geometry, $\sigma_{y}>\sigma_{z}$. In fact, this leads the uncertainty of the horizontal axis to contribute much more to the dispersion of the vertical displacements rather than the horizontal ones, thus introducing uncertainty in the retrieval of the angle. The concept is emphasized by the scatter plot in Figure 6, in which two examples of displacement with different directions are reported in the y−z plane. For the sake of clarity, $\sigma_{z}$ is considered to be zero in the plot.

Equations (7) and (9) make explicit the relation between the geometry and the uncertainties, thus providing a valuable tool to define the installation geometry when the monitoring of bridges with two radars is concerned.

### 2.4. Radar Equipment

**Figure 7:**
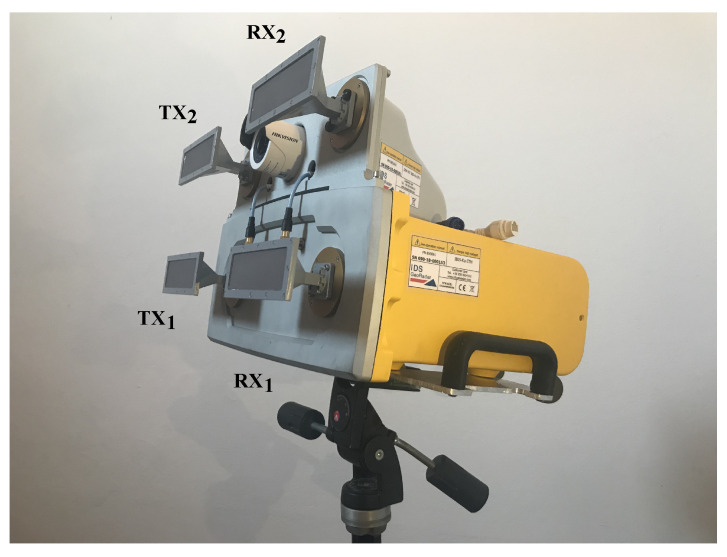
IBIS-FM Radar by IDS Georadar company.

The radar used in this work was a modified version of the IBIS-FM MIMO by IDS Georadar (Pisa, Italy) [34,41], and it is shown in Figure 7. It provides an FMCW signal with central frequency $f_{c}=17.2\;$GHz, bandwidth $B_{max}=400\;$MHz, and $N_{sample}=805$. With these parameters, the unambiguous range was about $R_{u}=2\cdot 10^{3}\;$m, the range resolution ΔR=c/(2·B)=1.5m, and the pulse repetition frequency PRF=740Hz.

The radar has two transmitting (TX) and two receiving (RX) channels, which operate sequentially: for each transmitted signal from the TX*_i_* channel, where i=1,2, the radar receives by means of the channel RX*_j_*, where j=1,2, thus obtaining four sequential acquisitions corresponding to the combinations ij=11,12,21,22, respectively. Despite its sequential operation, this device can be considered to operate as a MIMO radar in the context of bridge monitoring. In fact, the time it takes to perform the four acquisitions is $t_{acq}=4/PRF=5.4\;$ms, and since the natural frequencies of a bridge are of the order of a few Hz, the four channels can be considered synchronous between each other. The radar setup as shown in Figure 7 was used for the monostatic measurement.

**Figure 8:**
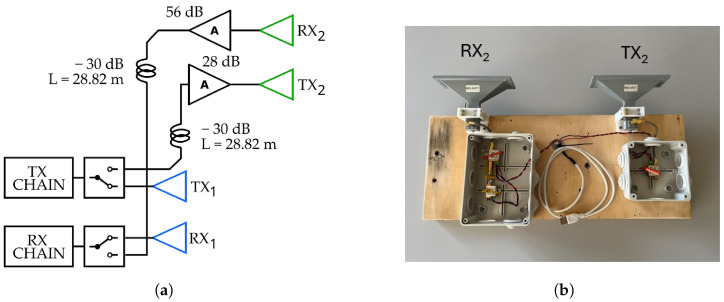
(**a**) Schematic setup of the IBIS-FM radar for the multimonostatic measurement; the combination in this case was ij=11. (**b**) Picture of the TX_2_–RX_2_ pair with the relative amplification chains.

With the aim of performing a multimonostatic measurement, the MIMO feature of the radar was exploited in such a way as to obtain two independent radars that were able to carry out simultaneous measurements from two different locations. This was achieved as explained in the following. The baseline was realized by separating the transmitting and receiving antennas of the radar. In particular, the TX_2_–RX_2_ pair was separated from the TX_1_–RX_1_ pair by means of two RF cables, as depicted in Figure 8. The TX_2_–RX_2_ pair was connected to a transponder, Figure 8, which is composed of a couple of antennas and the amplifiers.

The cables were ≈23 m long, of which the equivalent electric length was ≈28.8 m and the attenuation was −30 dB. To compensate for the cable attenuation, two amplifiers were added in the TX and RX channels of the transponder. The gain of TX amplifier was 28dB, and gain of RX amplifier was 56dB. The RX amplifier is a low-noise amplifier (LNA) with 5 dB of noise figure. The LNA was placed at the beginning of the receiving chain in order to minimize the total noise figure.

## 3. Results

As stated above, in this work two measurements were carried out: one in the monostatic configuration and a subsequent one in the multimonostatic configuration. The measurements lasted about an hour; during this hour, the bridge was subject to traffic stimulation. The weather conditions were stable, and there was a breeze. The environmental conditions were considered such as not to significantly influence neither the radar performance nor the measurement accuracy. The acquired data were analyzed by means of the commercial software MATLAB, version R2023a. A detailed description of the measurements setup and the relative results are provided in the following subsections.

### 3.1. Monostatic Measurement

**Figure 9:**
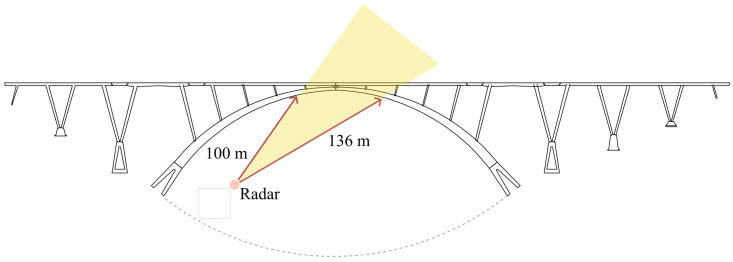
Schematic setup of the monostatic measurement for the monitoring of the Bisantis bridge.

**Figure 10:**
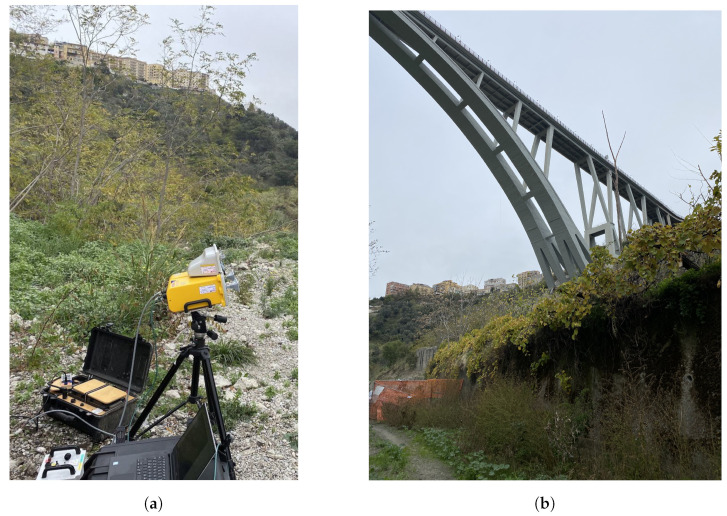
(**a**) Picture of the IBIS-FM while operating in the monostatic configuration. (**b**) Picture of the Bisantis bridge.

As mentioned above, the bridge considered in this work is arched; the horizontal deck is connected to the arch by some oblique uprights. A first monostatic measurement was carried out so as to capture the oblique uprights and the deck. The schematic of the setup is shown in Figure 9.The radar setup used in this measurement was the one shown in Figure 7. The radar was placed on a small hill under the bridge; the area separating it from the bridge was clear. It is worth mentioning that the schematic is in scale. In Figure 10a is shown a picture of the radar while operating in the monostatic configuration. In Figure 10b is shown a picture of the Bisantis bridge.

**Figure 11:**
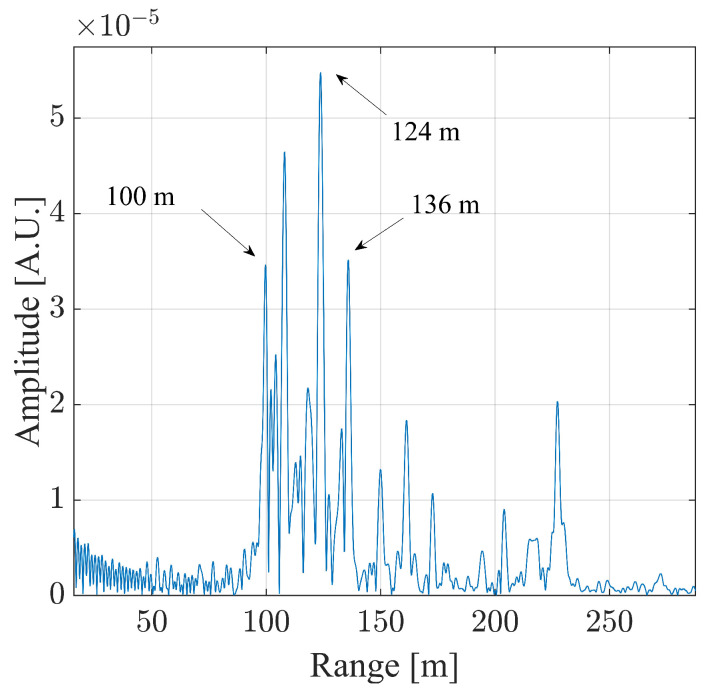
Point spread function resulting from the monostatic monitoring of the Bisantis bridge.

The Fourier transform of the measured data was calculated, with which the point spread function was obtained, as shown in Figure 11.

**Figure 12:**
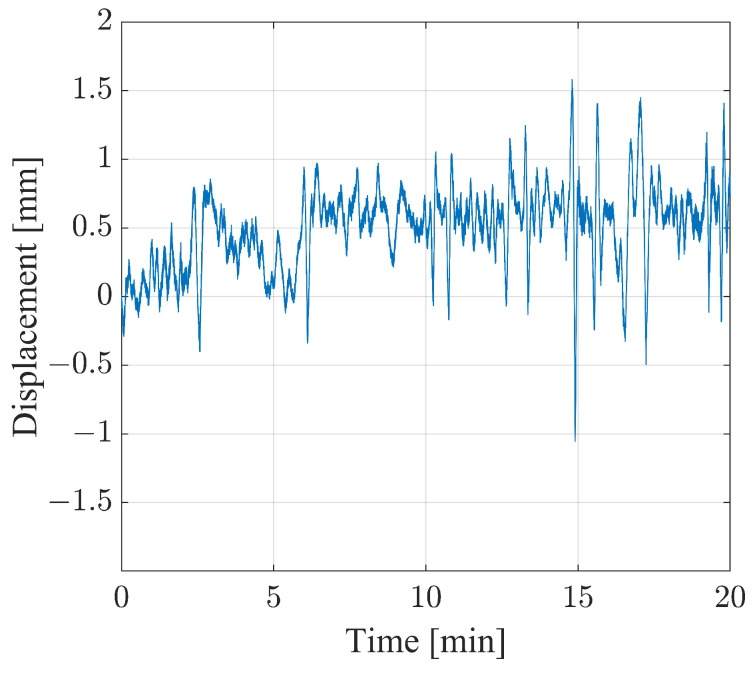
Displacement resulting from the monostatic monitoring of the Bisantis bridge.

The targets between 100m and 136m were analyzed. For the sake of brevity, the analysis of only one target is reported in this work. The highest one was considered, namely the one corresponding to 124m. As described in the works [22,26], the authors are aware that in the case that a transversal component was present, such a setup would introduce an interpretation error in the calculation of the vertical component. For this reason, the displacement shown in Figure 12 is reported in the radar line of sight. Generally, the measurement uncertainty of an interferometric radar is lower than 0.1mm [42]. Thus, under the assumption of an uncertainty of 0.1mm affecting the radar used in this work, this coincides with the uncertainty affecting the displacement module. The displacement module reaches its maximum approximately in the 15th min, thus exceeding 2.5mm. Other significant events occurred approximately at the minutes 2.7,15.5,16.8, and 19.5, at which the module reached 1.1mm, 1.6mm, 1.9mm, and 1.6mm, respectively.

**Figure 13:**
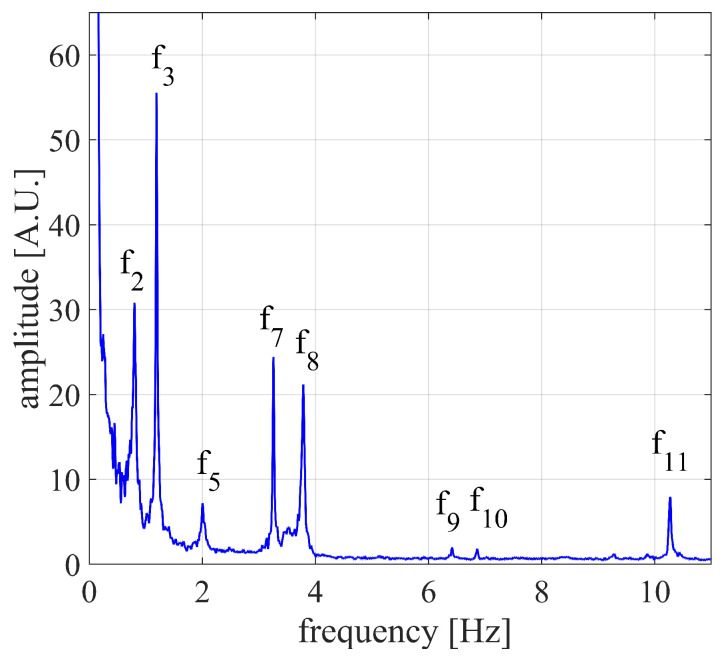
Natural frequancies resulting from the monostatic monitoring of the Bisantis bridge.

The JTFA [40] of the displacement was performed, and the results are reported in Figure 13. The frequencies identified were about eight in all, which are listed in Table 1 (the frequencies were numbered with reference to the results shown in the following subsection).

### 3.2. Multimonostatic Measurement

**Figure 14:**
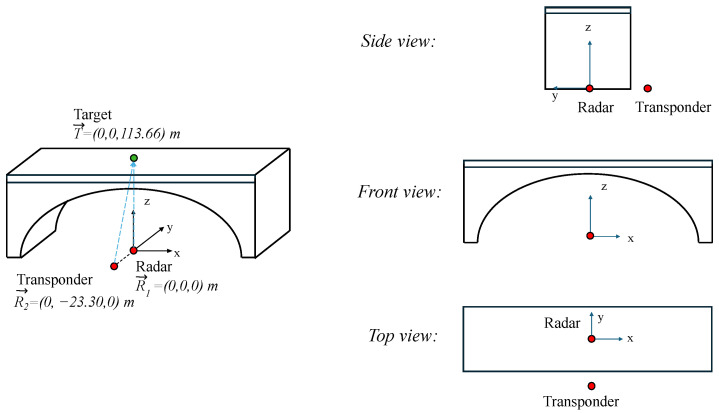
Schematic setup for the multimonostatic monitoring of the Bisantis bridge.

**Figure 15:**
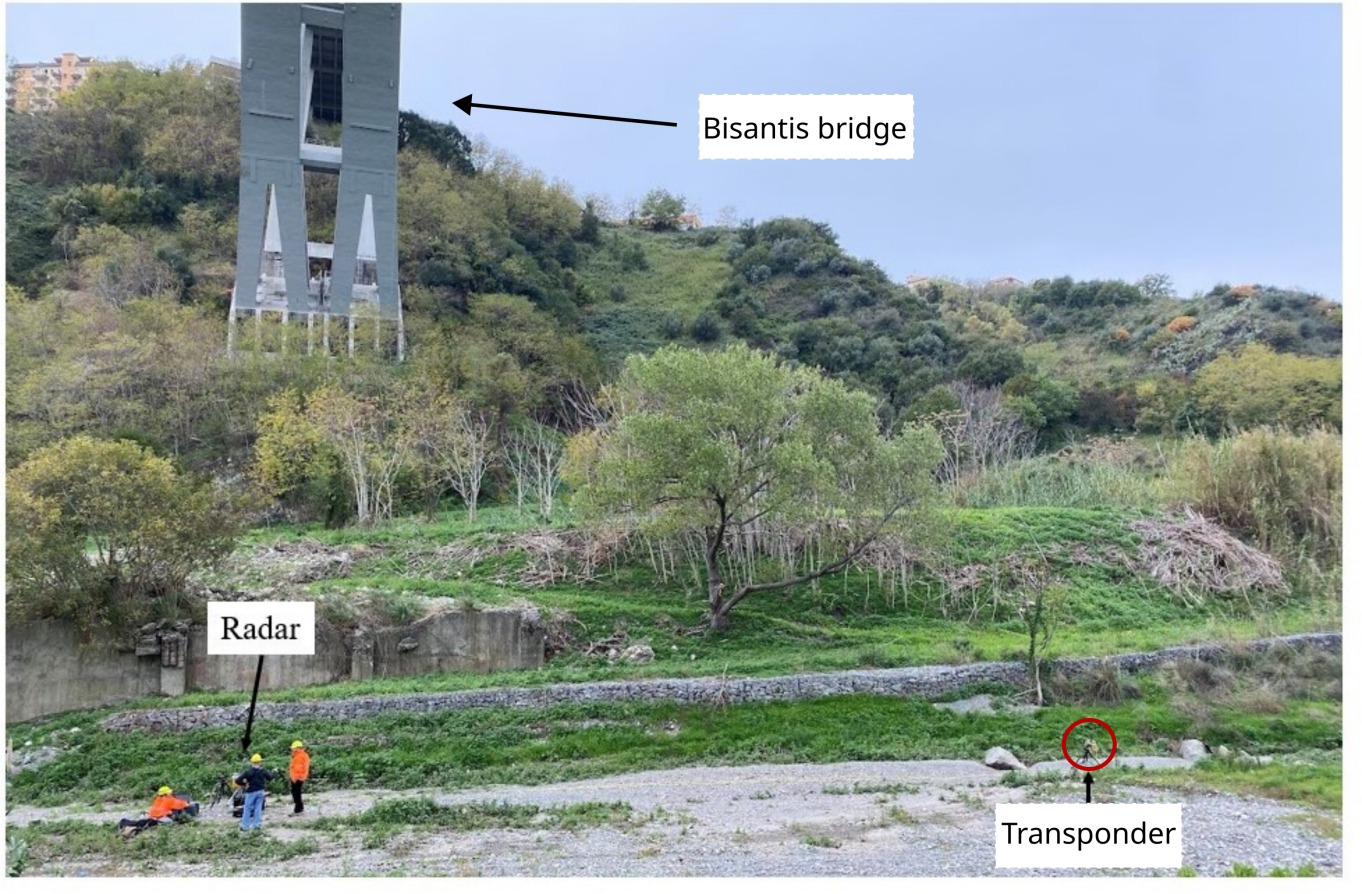
Picture of the experimental setup for the multimonostatic monitoring of the Bisantis bridge. The picture was taken from the side view.

The radar setup used for this measurement was the one depicted in Figure 8a. In Figure 14 and Figure 15, the schematic and the picture of the adopted setup for the detection of the displacement components are shown, respectively. The radar was placed approximately at the center of the span, and it was aimed in the vertical direction. The transponder was placed approximately 20m away from the radar in the direction of the *y* axis. The transponder was aimed at the same point on the deck. The rational was to measure two components $\Delta R_{1}$ and $\Delta R_{2}$ along the directions of $\vec{T}-\vec{R_{1}}$ and $\vec{T}-\vec{R_{2}}$, respectively.

**Figure 16:**
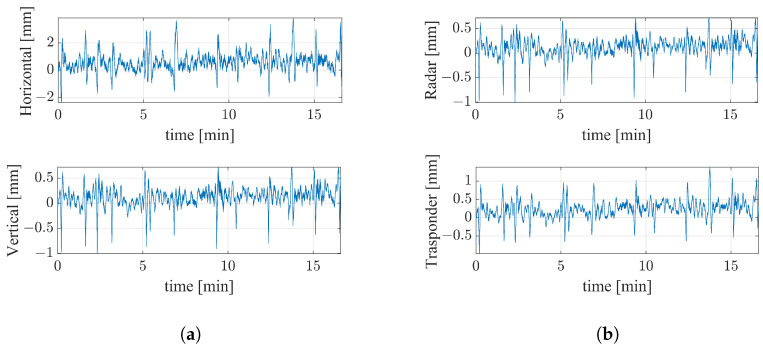
(**a**) Displacements along the horizontal and vertical directions. (**b**) Displacements along the directions $\vec{T}-\vec{R_{1}}$ (radar) and $\vec{T}-\vec{R_{2}}$ (transponder), respectively.

The displacement components of the bridge were calculated by means of Equation (6). The cumulative displacements were then computed and reported in Figure 16 as functions of time. The horizontal component results were much larger than the vertical ones, as is evident from Figure 16a. In particular, the first exceeded 4mm approximately at the minutes 0.2, 1.7, 6.8, 12.4, 13.8, and 15, while the second remained bounded in ≈1.5 mm during the entire measurement. In Figure 16b are reported the displacements detected by the radar and the transponder.

As already mentioned in the previous subsection, generally the measurement uncertainty of an interferometric radar is lower than 0.1mm. Thus, under the assumption that the radar and the transponder operated under the same condition with respect to the signal-to-noise ratio (SNR) ($\sigma_{R_{1}}=\sigma_{R_{2}}$), the Equation (7) provided us the uncertainties on Δy and Δz, thus resulting in ≈0.7 mm and ≈0.1 mm, respectively.

**Figure 17:**
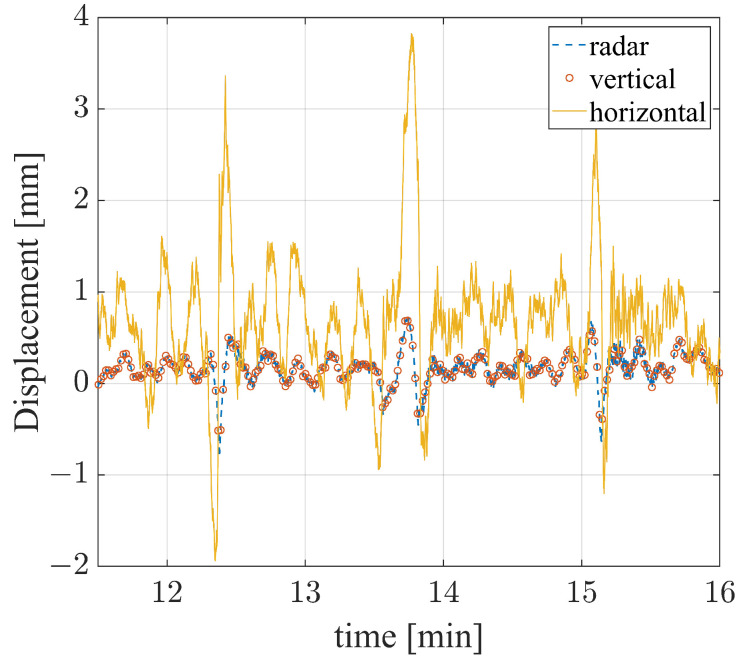
Comparison between the displacements retrieved from the single measurement (radar) and the double measurement (vertical and horizontal).

To better appreciate the contribution of the multimonostatic approach with respect to the standard monostatic one, in Figure 17 are reported the displacement values retrieved from the single measurement, namely the radar, and the ones retrieved from the double measurements, namely the vertical and the horizontal measurements. The data were reported from about the 11th min up to the 16th min for better clarity of the comparison. It is worth noting that, given the adopted geometry, the displacement retrieved from the radar coincides with the vertical displacement retrieved from the double measurement radar–transponder. The horizontal displacement results were about four times higher than the vertical ones.

Without the transponder, the dynamic description of the bridge would lack its major contributing displacement component.

**Figure 18:**
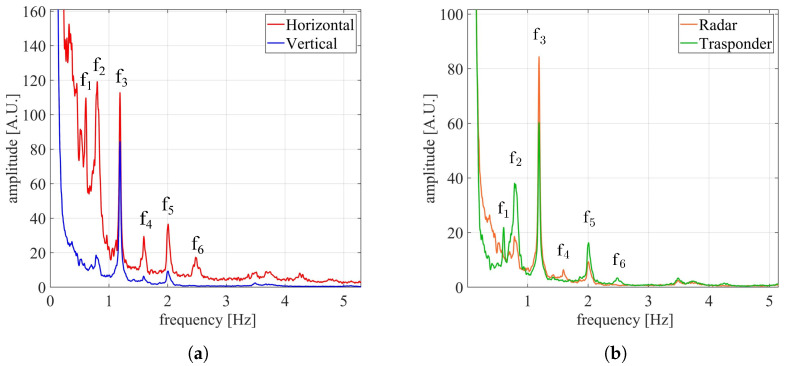
(**a**) JTFA computed on the displacements occurring along the horizontal (red) and vertical (blue) directions, respectively. (**b**) JTFA computed on the displacements occurring along the directions $\vec{T}-\vec{R_{1}}$ (orange) and $\vec{T}-\vec{R_{2}}$ (green), respectively.

In order to obtain the natural frequencies of the bridge, the JTFA of the displacements was performed, and the results are reported in Figure 18. In Figure 18a, the results of the JTFA computed for the the horizontal and vertical displacements are shown, while in Figure 18b, the results of the JTFA computed on the displacements along the radar and the transponder directions are shown. The natural frequencies were six in total, namely $f_{1},f_{2},f_{3},f_{4},f_{5},\;\mathrm{and}\;f_{6}$, whose values are reported in Table 2. For comparison purposes, the frequencies resulting from the monostatic measurement were also reported.

The results of Figure 18a show that, as expected, all of the six detected natural frequencies had an horizontal component larger than the vertical one. In particular, it is worth noting that the horizontal component was dominant in almost all of the displacements. The results reported in Figure 18b show that, as expected, the radar only identified the frequencies with a vertical component, namely $f_{2},f_{3},f_{4},\;\mathrm{and}\;f_{5}$. Instead, the transponder identified five frequencies, namely $f_{1},f_{2},f_{3},f_{5},\;\mathrm{and}\;f_{6}$. The reason why $f_{4}$ was not detected by the transponder is related to the displacement direction, as explained in next subsection.

A couple of observations concerning the comparison between the two configurations are needed. The higher frequencies detected from the monostatic measurement are probably related to the oblique uprights. In fact, the measurement was carried out obliquely to the bridge, which was thus capable of capturing the underlying structure, as well as the deck. Instead, the multimonostatic measurement was carried out vertically, with both the radar and the transponder aiming at the center of the deck, thereby capturing an area without the underlying structure. Furthermore, since the monostatic measurement was carried out under the bridge, the detection of the displacements with a vertical component was expected. By looking at Table 2, such displacements detected in the multimonostatic configuration were four, namely $f_{2},f_{3},f_{4},$ and $f_{5}$. However, the monostatic configuration detected $f_{2},f_{3},$ and $f_{5}$. This could be explained by the fact that the radar was placed obliquely to the bridge, thus retrieving the projection of the vertical component, which as such is smaller than the actual one. This hypothesis is supported by the fact that, among the displacements with a vertical component, $f_{4}$ is the smallest one, as is evident from Figure 18a. Moreover, since the two measurements were carried out at different times, it is possible that such a weak frequency was not excited during the first measurement.

### 3.3. Displacement Direction Retrievement

Deep insights into the multimonostatic results are provided by retrieving the displacements directions. A significant time period was selected on the displacement data. On the data were applied six band-pass Butterworth filters with 15 poles, with each centered on a specific natural frequency $f_{i}$, where i=1,…,6; the results were reported as scatter plots in the y–z plane. A linear fit was carried out for each of the six groups of data, and the angle α was retrieved from the angular coefficient of the fitting lines. A potential source of errors in this evaluation is given by the presence of different displacement directions overlapped between themselves. However, in this work, the main purpose of this procedure was to highlight the preferential tendency of most of the displacements, thus providing more concreteness to the results of the previous subsections.

**Figure 19:**
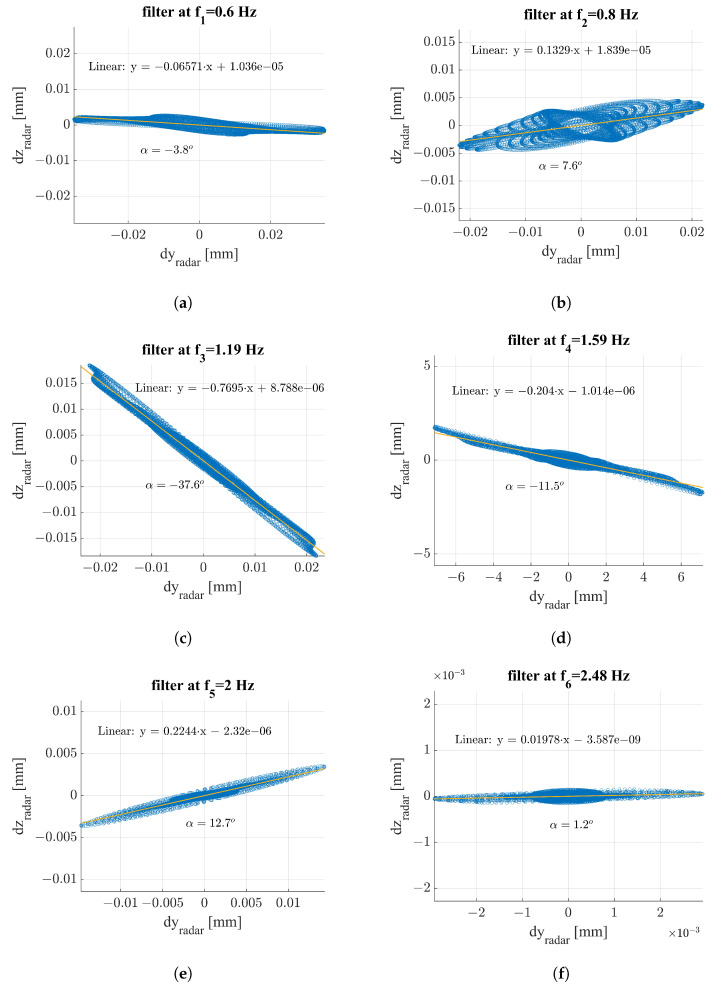
Oscillation direction corresponding to each frequency detected on the Bisantis bridge: (**a**) $f_{1}=0.6\;$Hz, (**b**) $f_{2}=0.8\;$Hz, (**c**) $f_{3}=1.19\;$Hz, (**d**) $f_{4}=1.59\;$Hz, (**e**) $f_{5}=2.00\;$Hz, and (**f**) $f_{6}=2.49\;$Hz.

The results are shown in Figure 19. In Table 3 are reported the directions in degrees of each frequency. It is worth noting that the displacement directions were retrieved only on the multimonostatic measurement, since two components are required. From a first look at Figure 19, one can appreciate that all the natural frequencies were characterized by a displacement mainly occurring in the horizontal direction. In particular, Figure 19a,b,d–f show that the displacements with frequencies $f_{1},f_{2},f_{4},f_{5},$ and $f_{6}$ respectively generated an angle in absolute value of less than 15° with the horizontal axis. Furthermore, from Figure 19, we can assess that the displacement with frequency $f_{4}$ occurred in a perpendicular direction with respect to $\vec{T}-\vec{R_{2}}$; in fact, the angle α≈11.5° was coincident with the one between $\vec{T}-\vec{R_{2}}$ and the z axis. This is the reason why $f_{4}$ was not detected by the transponder in the multimonostatic measurement.

## 4. Discussion

Based on the results of the monostatic measurement, the natural frequencies greater than 3Hz are probably related to the oblique uprights. This could be considered to be the main contribution made by the monostatic measurement to the overall analysis. In addition, given that the monostatic measurement is a standard in the context of bridge monitoring, it could act as a reference for future monitoring of the Bisantis bridge. In fact, while from one side the geometry adopted in this work led the vertical displacement module to be affected by an interpretation error, from the other side, the retrievement of the frequencies of the vertical displacements is expected to be correct.

Based on the results of the multimonostatic measurement, it is evident that the movement of the bridge under test mainly occurred along the transversal direction. This fact highlights the importance of using at least two radars for retrieving a correct description of the displacement vector.

It should be noted that, in terms of data collection and analysis, the monostatic and multimonostatic configurations are identical; obviously, in order to retrieve the vertical and horizontal components, the multimonostatic configuration requires the prior calculation of the rotation matrix.

A discussion concerning the multimonostatic measurement setup adopted in this work and the advices for future operators in this field are provided in the following. From one side, the simplicity of the setup implemented in this work allowed the authors to perform the whole measurement campaign in less than an hour. From the other side, given that H/B≈5, the uncertainty of Δy was about seven times the one of Δz, as one can deduce from Figure 4; the limitation was represented by the RF cable lengths. Therefore, in the case of high bridges and for which a transversal displacement is expected, the ideal case would be to locate the radar and the transponder in such a way as to make their view directions as much as possible perpendicular between each other. This could be achieved by means of a radio link, but at the cost of a considerable effort in terms of complexity and installation time. However, the setup adopted in this work allowed the authors to retrieve the displacement direction in an accurate way. In fact, with the displacement directions being mainly horizontal, the presence of the radar under the bridge minimized $\sigma_{\alpha }/\sigma_{R}$, as is evident from Figure 5.

The novelty of the work relies in two different aspects: (1) it provides closed-form formulas for the estimation of the measurement uncertainty in dependence on the multimonostatic geometry setup, and (2) it shows the performance of the multimonostatic approach in the monitoring of a bridge whose dominant displacement component is the horizontal one.

Finally, the authors observe that in the work of [39], the main natural frequency of the Bisantis bridge was ≈5 Hz, while in the present work, it was $f_{3}\approx 1.19\;$Hz. The difference in this evaluation could be attributed to the different technique adopted, since the data were retrieved from satellite images and from interferometric measurements. The authors believe that the use of phase information is in general a reliable method for the calculation of the natural frequencies of bridges.

## 5. Conclusions

In this work, two different measurements were carried out on an arched bridge. In the first one, a monostatic measurement able to retrieve the frequencies of the oblique uprights was carried out. Then, a multimonostatic MIMO technique was used for the detection of the displacement components.

In order to provide a rationale in the choice of the geometry for the multimonostatic measurement, analytical formulas concerning the calculation of the uncertainty, for both the displacement and the oscillation direction, were provided. The multimonostatic measurements revealed that the bridge moved almost totally along the horizontal axis; the technique was able to retrieve the displacement along this direction, thus showing values exceeding 4mm. Six natural frequencies of the bridge were determined, and the preferential direction was retrieved, thereby confirming the horizontal movement of the bridge. The present study highlights the importance of retrieving at least two displacement components in the context of bridge monitoring.

## Figures and Tables

**Table 1:** Frequencies of the displacement detected on the Bisantis bridge by means of the monostatic measurement.

	[Hz]
$f_{2}$	0.8
$f_{3}$	1.19
$f_{5}$	2
$f_{7}$	3.26
$f_{8}$	3.8
$f_{9}$	6.42
$f_{10}$	6.86
$f_{11}$	10.27

**Table 2:** Frequencies of the horizontal and vertical components of the displacements detected on the Bisantis bridge by means of the multimonostatic measurement. For comparison, the frequencies resulting from the monostatic measurement were also reported.

	Multimonostatic (Horizontal) [Hz]	Multimonostatic (Vertical) [Hz]	Monostatic [Hz]
$f_{1}$	0.60	-	-
$f_{2}$	0.80	0.80	0.80
$f_{3}$	1.19	1.19	1.19
$f_{4}$	1.59	1.59	-
$f_{5}$	2.00	2.00	2.00
$f_{6}$	2.48	-	-
$f_{7}$	-	-	3.26
$f_{8}$	-	-	3.80
$f_{9}$	-	-	6.42
$f_{10}$	-	-	6.86
$f_{11}$	-	-	10.27

**Table 3:** Oscillation direction, in degrees, corresponding to each frequency detected on the Bisantis bridge.

	α [DEG]
$f_{1}$	−3.8°
$f_{2}$	7.6°
$f_{3}$	−37.6°
$f_{4}$	−11.5°
$f_{5}$	12.7°
$f_{6}$	1.2°

## Data Availability

The data presented in this study are available on request from the corresponding author due to privacy.
